# Stafne Bone Cyst: A Report of an Unusual Case

**DOI:** 10.7759/cureus.83780

**Published:** 2025-05-09

**Authors:** Asish K Das, Aritra Chatterjee, Purbalee Barman, Abhijit Maji, Nayana De

**Affiliations:** 1 Oral and Maxillofacial Surgery, Dr. R. Ahmed Dental College and Hospital, Kolkata, IND

**Keywords:** lingual mandibular bone defect, mandibular angle, stafne bone cyst, stafne defect, unilocular radiolucency

## Abstract

Stafne bone cyst (SBC) is a rare, asymptomatic finding typically located in the posterior region of the mandible, often observed incidentally on radiographs. The diagnosis of the defect is usually made using orthopantomogram, but in uncommon cases, more precise imaging techniques such as cone-beam computed tomography (CBCT), computed tomography (CT), and magnetic resonance imaging (MRI) are required. It is a well-circumscribed radiolucency that represents a developmental bone defect rather than a true cyst. This case report discusses a rare presentation of Stafne’s bone cyst in a 30-year-old male patient, who also had concurrent pericoronitis. The radiograph incidentally showed a radiolucency below the mandibular canal in the left angle region of the mandible.

## Introduction

Stafne's bone defect is a rare mandibular deformity, first described by Edward C. Stafne in 1942 [1]. Since the patients frequently do not exhibit any unusual clinical symptoms, it is typically discovered by chance on radiographs taken during routine dental procedures [2]. An oval or spherical radiolucency can be seen on panoramic radiography near the mandibular angle, typically situated between the inferior alveolar canal and the lower edge of the mandible. The size of the lesion ranges from 0.3 cm to 8 cm, with a mean of 1.58 cm [3]. Stafne bone cyst is easy to diagnose with CT and MRI; however, it is necessary to perform a differential diagnosis with other cysts and tumor-like lesions in the mandible, such as ameloblastoma, residual cyst, periapical cyst, metastatic lesions, or salivary gland lesions [4]. Thus, it is essential to properly diagnose any lesion related to the mandibular angle region, positioned below the inferior alveolar canal, with the corroboration of clinical and radiological evidence to avoid misdiagnosis and unnecessary surgical intervention.

## Case presentation

**Figure 1:**
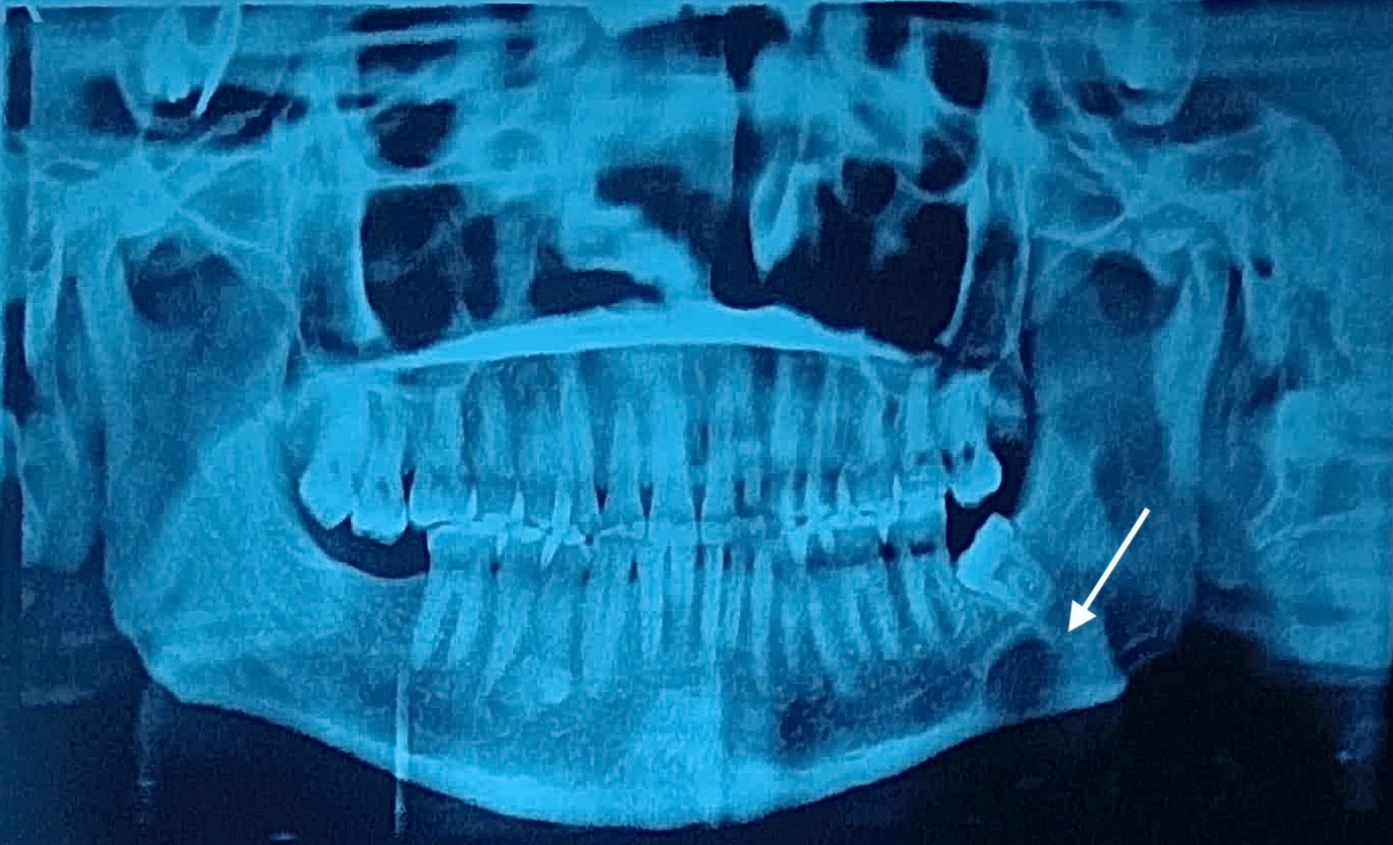
Orthopantomogram of the patient (the white arrow shows the lesion)

**Figure 2:**
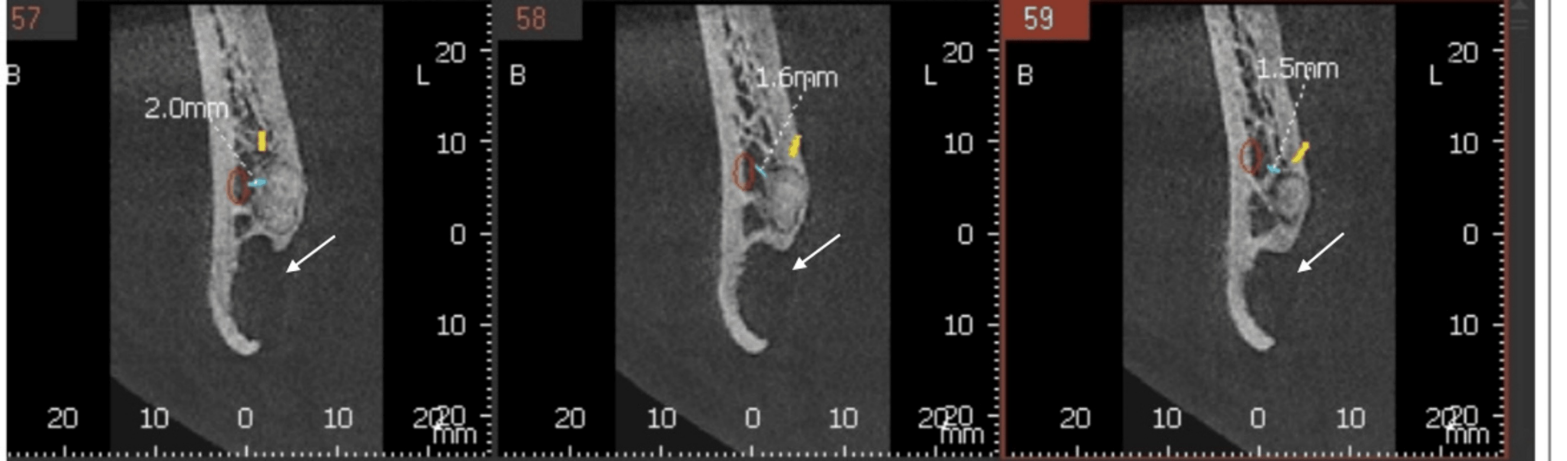
Cone-beam computed tomography of the patient (white arrows show the Stafne cyst)

**Figure 3:**
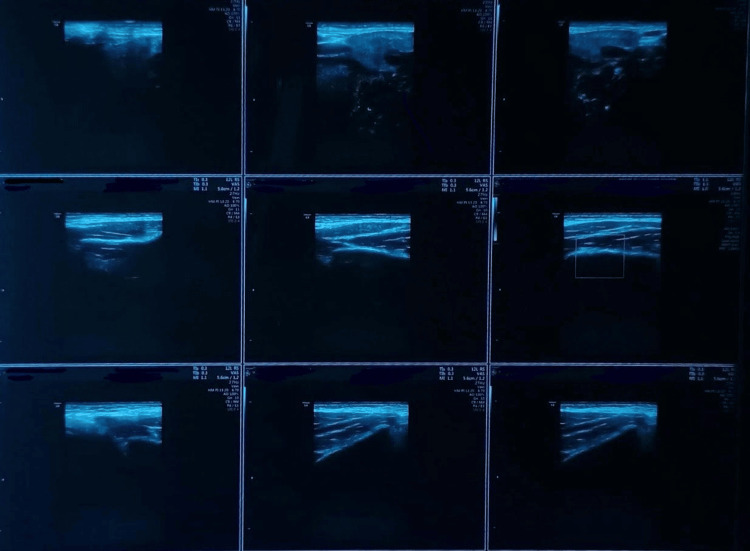
Colour Doppler study of left angle of mandible

A 30-year-old male reported to the Department of Oral and Maxillofacial Surgery with a complaint of pain in his left lower jaw. Earlier, the patient had consulted a private clinician for the same. The patient was advised a panoramic radiograph (Figure 1). He was found to have a round radiolucent lesion in the left lower jaw for which he was advised to undergo immediate surgical intervention. The anxious patient reported to our department with the specific need of having the lesion removed. He was having acute pain associated with the same region. During intraoral examination, mild swelling, redness, and slightly reduced mouth opening were observed distal to the last teeth in the left lower jaw. No significant medical or family history was given by the patient. He was in possession of his panoramic radiograph, which had been advised earlier. That radiograph revealed an erupting mesially tilted third molar in the left lower quadrant and a well-defined, unilocular radiolucent lesion measuring approximately 2 cm in diameter, situated beneath the erupting tooth and also below the inferior alveolar canal. No signs of cortical expansion or root resorption were observed. To rule out any vascular lesion of the jaw, we advised a color Doppler study of the left angle region of the mandible. Additionally, a cone-beam computed tomography (CBCT) was done (Figure 2), which revealed the presence of a concavity on the lingual surface of the mandible. The color Doppler study (Figure 3) ruled out the presence of a vascular lesion in the said area. Faced with a diagnostic dilemma, we advised an ultrasound-guided fine needle aspiration cytology (FNAC) of the lesion. Cytology studies confirmed the presence of numerous salivary acini in the lesion and ruled out any tumor cells in the same. The diagnosis now definitively leaned towards a Stafne’s bone cyst, and the pain that the patient had been experiencing was more likely a result of pericoronitis in relation to the left lower third molar, and the innocuous Stafne’s cyst had no role in his misery.

Having reassured the patient and gained his confidence, we proceeded with a simple disimpaction surgery of the concerned third molar. The patient was placed on follow-up and, needless to say, he experienced a complete resolution of his symptoms. Thus, we not only prevented overtreatment by a detailed and correct diagnosis but also ensured that the patient went home free of anxiety. The patient is kept under regular follow-up at six-month intervals. Currently, he is free of any symptoms, one year since the intervention.

## Discussion

Edward Stafne first described the Stafne bone cyst in 1942. According to him, it appears as a radiolucent cavity that can frequently be observed unilaterally underneath the inferior alveolar nerve canal and approximately above the inferior border of the mandible [1]. After Stafne, other authors also described similar cavities, which were mainly observed in men within the age group between 50 and 70 years, with a prevalence rate of 0.10%-0.48% [5].

Despite the uncertain etiology, the most widely accepted pathogenesis is the "glandular" hypothesis [2]. According to this theory, the lesion arises from compression of the lingual surface of the mandible, especially due to the sublingual gland, followed by resorption of the lingual cortical plate, finally resulting in a depression or defect on the lingual aspect of the mandible [6,7]. Given the findings obtained, Choukas et al., in 1960, proposed that the Stafne bone cyst could potentially be caused by embryonic entrapment of the submandibular gland's lobes or due to unknown exertion from a hypertrophied submandibular gland [8]. Lello and colleagues proposed a theory that the defect develops due to relative ischemia [9]. The authors described that the mandibular lingual cortex is compressed in an area near the passage of the facial artery, and the lesion arises as a result of poor blood flow to the cortex due to a combination of superiorly and medially directed tensile muscle and hemodynamic forces acting on the facial artery, pulling it away from the lingual cortex and thereby compromising the nutrition of the cortex [3]. Three age-associated investigations were conducted by D'Eramo et al., who hypothesized that the cyst was developmental [10].

The histopathology of the Stafne bone cyst has reported the absence of any cystic lesion but the presence of mixed salivary gland tissue along with normal sublingual gland [5]. Diseases that must be diagnosed differentially from the Stafne bone cavity include cysts such as odontogenic cyst, traumatic bone cyst, periapical cyst, residual cyst, and tumors such as ameloblastoma, giant cell tumor, brown tumor, as well as nonossifying fibroma, vascular malformation, and fibrous dysplasia [5].

A conservative approach is preferred over any surgical intervention. Since the Stafne bone cyst is a benign, asymptomatic, developmental bony defect without any pathological changes, surgical treatment is usually not required. However, regular follow-ups are recommended to observe the growth of the cyst and to prevent its progression [11].

## Conclusions

Stafne bone cyst is a rare benign mandibular defect that is often discovered incidentally. Accurate diagnosis through imaging is crucial to avoid unnecessary surgical procedures. This case highlights the importance of proper diagnosis of the Stafne bone cyst as a developmental anomaly with no need for intervention, along with identifying the correct cause. However, periodic follow-ups must be performed at regular intervals to monitor the lesion and prevent its progression.

## References

[1] Stafne EC (1942). Bone cavities situated near the angle of the mandible. J Am Dent Assoc.

[2] Chen MH, Kao CT, Yu-Fong Chang J, Wang YP, Wu YH, Chiang CP (2019). Stafne bone defect of the molar region of the mandible. J Dent Sci.

[3] Soares A, Ferreira L, Calderipe C (2023). Stafne's bone defect: a systematic review. Med Oral Patol Oral Cir Bucal.

[4] Liang J, Deng Z, Gao H (2019). Stafne's bone defect: a case report and review of literatures. Ann Transl Med.

[5] Taysi M, Ozden C, Cankaya B, Olgac V, Yıldırım S (2014). Stafne bone defect in the anterior mandible. Dentomaxillofac Radiol.

[6] Philipsen HP, Takata T, Reichart PA, Sato S, Suei Y (2002). Lingual and buccal mandibular bone depressions: a review based on 583 cases from a world-wide literature survey, including 69 new cases from Japan. Dentomaxillofac Radiol.

[7] Schneider T, Filo K, Locher MC (2014). Stafne bone cavities: systematic algorithm for diagnosis derived from retrospective data over a 5-year period. Br J Oral Maxillofac Surg.

[8] Choukas NC (1973). Developmental submandibular gland defect of the mandible: review of the literature and report of two cases. J Oral Surg.

[9] Lello GE, Makek M (1985). Stafne's mandibular lingual cortical defect. Discussion of aetiology. J Maxillofac Surg.

[10] D'Eramo EM, Poidmore SJ (1975). Developmental submandibular gland defect of the mandible. Review of the literature and report of a case. Oral Surg Oral Med Oral Pathol.

[11] Aoki EM, Abdala-Júnior R, Nagano CP, Mendes EB, Oliveira JX, Lourenço SV, Arita ES (2018). Simple bone cyst mimicking Stafne bone defect. J Craniofac Surg.

